# Cortical thickness modeling and variability in Alzheimer’s disease and frontotemporal dementia

**DOI:** 10.1007/s00415-023-12087-1

**Published:** 2023-11-27

**Authors:** Agnès Pérez-Millan, Sergi Borrego-Écija, Neus Falgàs, Jordi Juncà-Parella, Beatriz Bosch, Adrià Tort-Merino, Anna Antonell, Nuria Bargalló, Lorena Rami, Mircea Balasa, Albert Lladó, Roser Sala-Llonch, Raquel Sánchez-Valle

**Affiliations:** 1 Alzheimer’s Disease and Other Cognitive Disorders Unit. Service of Neurology, Hospital Clínic de Barcelona. Fundació Recerca Clínic Barcelona-IDIBAPS, Villarroel, 170, 08036 Barcelona, Spain; 2 Institut de Neurociències, University of Barcelona, 08036 Barcelona, Spain; 3 Department of Biomedicine, Faculty of Medicine, University of Barcelona, 08036 Barcelona, Spain; 4 Atlantic Fellow for Equity in Brain Health, Global Brain Health Institute, University of California San Francisco, San Francisco, 94143 USA; 5 Image Diagnostic Centre, CIBER de Salud Mental, Instituto de Salud Carlos III, Magnetic Resonance Image Core Facility, IDIBAPS, Hospital Clínic de Barcelona, Barcelona, Spain; 6 Centro de Investigación Biomédica en Red de Bioingeniería, Biomateriales y Nanomedicina (CIBER-BBN), 08036 Barcelona, Spain; 7 Departament de Medicina, Facultat de Medicina i Ciències de la Salut, Universitat de Barcelona, 08036 Barcelona, Spain

**Keywords:** Alzheimer’s disease, Frontotemporal dementia, Magnetic resonance imaging, Cortical thickness, Predictive modeling

## Abstract

**Background and objective:**

Alzheimer’s disease (AD) and frontotemporal dementia (FTD) show different patterns of cortical thickness (CTh) loss compared with healthy controls (HC), even though there is relevant heterogeneity between individuals suffering from each of these diseases. Thus, we developed CTh models to study individual variability in AD, FTD, and HC.

**Methods:**

We used the baseline CTh measures of 379 participants obtained from the structural MRI processed with FreeSurfer. A total of 169 AD patients (63 ± 9 years, 65 men), 88 FTD patients (64 ± 9 years, 43 men), and 122 HC (62 ± 10 years, 47 men) were studied. We fitted region-wise temporal models of CTh using Support Vector Regression. Then, we studied associations of individual deviations from the model with cerebrospinal fluid levels of neurofilament light chain (NfL) and 14–3-3 protein and Mini-Mental State Examination (MMSE). Furthermore, we used real longitudinal data from 144 participants to test model predictivity.

**Results:**

We defined CTh spatiotemporal models for each group with a reliable fit. Individual deviation correlated with MMSE for AD and with NfL for FTD. AD patients with higher deviations from the trend presented higher MMSE values. In FTD, lower NfL levels were associated with higher deviations from the CTh prediction. For AD and HC, we could predict longitudinal visits with the presented model trained with baseline data. For FTD, the longitudinal visits had more variability.

**Conclusion:**

We highlight the value of CTh models for studying AD and FTD longitudinal changes and variability and their relationships with cognitive features and biomarkers.

**Supplementary Information:**

The online version contains supplementary material available at 10.1007/s00415-023-12087-1.

## Introduction

During the last two decades, the study of several biomarkers, including neuroimaging, has substantially improved the diagnosis of neurodegenerative dementias. However, there’s still a need for reliable biomarkers to track disease, evaluate the effect of experimental drugs, or provide an accurate prognosis. Both Alzheimer’s disease (AD) and frontotemporal dementia (FTD) are characterized by prototypical clinical features and patterns of progressive brain atrophy that constitute the disease’s fingerprint or signature. However, both diseases show relatively high individual heterogeneity in presentation and progression rate. The study of this variability is relevant to better understanding these diseases’ pathogenesis and predicting disease progression and potentially the effect of treatments [1]. Previous studies also suggest that the age of onset might influence the longitudinal evolution of AD patients [2, 3], emphasizing the need to model age and time from symptoms onset.

Quantitative neuroimaging studies with Magnetic Resonance Imaging (MRI) have been widely used to detect brain changes across these neurodegenerative disorders, using measures such as the cortical thickness (CTh) [4–6], but mostly in research studies. Cerebrospinal fluid (CSF) biomarkers, such as the amyloid-beta protein 42 (Aβ42), the total tau (t-tau), and phosphorylated tau (p-tau), have been included in the current criteria for AD diagnosis [7, 8]. Other CSF biomarkers, such as neurofilament light chain (NfL) levels, a marker of neuroaxonal damage, and 14-3-3 protein levels, a marker of synaptic-neuronal loss, have been both proposed as nonspecific neurodegeneration markers [9–12].

Modeling approaches that account for time are of paramount importance to understanding disease progression and comparing brain status across subjects at different disease stages [13–16]. Using structural MRI, some authors described CTh loss with time, providing valuable information on the characterization of disease trajectories and validation of prognostic biomarkers [17, 18].

We hypothesized that dementia is characterized by high variability in atrophy patterns reflecting clinical and biological differences across subjects. In this prospective study with 379 subjects, we aimed to develop CTh models for each diagnosis group (AD, FTD, and healthy controls (HC)), considering time from disease onset. We further aimed to study individual variability with respect to the model and evaluate the effect of the time from disease onset, cognition, and biochemical markers in the individual deviations from the model. Finally, we aimed to test these pseudo-longitudinal models in predicting the real longitudinal CTh evolution of a subsample of subjects.

## Materials and methods

### Participants

The prospective study includes 379 participants recruited from the Alzheimer's disease and other cognitive disorders group of the Hospital Clínic de Barcelona (HCB), Barcelona, Spain. The study was approved by the HCB Ethics Committee (HCB 2019/0105), complied with the Declaration of Helsinki, and all participants gave written informed consent. All participants underwent a complete clinical and cognitive evaluation [3, 19] and at least one 3T high-resolution structural MRI scan. A subset of these participants had novel CSF measures. A total of 309 participants had available CSF-NfL levels, and 160 participants had available CSF-14-3-3 measures. Additionally, a subset of 144 subjects underwent a second MRI scan 2 years after the baseline and 58 subjects with 4 years after the baseline scan. Participants with a history of stroke, traumatic brain injury, major psychiatric disorder, or alcohol abuse were excluded. Participants were classified into three groups:

- AD: participants meeting criteria for MCI or mild dementia due to AD [7, 8] supported by CSF biomarkers profile suggesting underlying AD neuropathology according to National Institute on Aging/Alzheimer's Association Research Framework 2018 [20].
- FTD: patients who met diagnostic criteria for either behavioral variant frontotemporal dementia (bvFTD) or FTD-related primary progressive aphasia (PPA) phenotypes, including Semantic Variant Primary Progressive Aphasia (svPPA) and Nonfluent Variant Primary Progressive Aphasia (nfvPPA) [21, 22]. All FTD patients included here showed a CSF profile not suggestive of AD.
- HC: healthy adults having cognitive performance within the normative range (cutoff 1.5 SD from the normative mean).

### CSF biomarkers

Commercially available single-analyte enzyme-linked immunosorbent assay (ELISA) kits were used to determine levels of CSF-NfL (IBL International, Hamburg, Germany) and 14-3-3 (CircuLex, MBL International Corporation, Woburn, MA) at the Alzheimer's disease and other cognitive disorders group laboratory, Barcelona, Spain.

### MRI acquisition

A high-resolution 3D structural dataset (T1-weighted, MP-RAGE, repetition time = 2.300 ms, echo time = 2.98 ms, 240 slices, field-of-view = 256 mm, voxel size = 1 × 1 × 1 mm) was acquired for everyone at each time point in a 3T Magnetom Trio Tim scanner (Siemens Medical Systems, Germany) upgraded to a 3T Prisma scanner (Siemens Medical Systems, Germany) during the study.

### MRI processing

We used FreeSurfer version 6.0 (http://surfer.nmr.mgh.harvard.edu.sire.ub.edu/) to perform cortical reconstruction of the T1-weighted acquisitions [23–25]. FreeSurfer allowed us to generate automated CTh maps for the left and the right hemispheres and to obtain summary measures within regions. We used the 68 cortical parcellations derived from the Desikan atlas available in FreeSurfer [26].

### CTh models 

We used the CTh values at baseline to generate three CTh models over time using Support Vector Regression (SVR). For the time variable, we used the chronological age of the subjects for HC subjects and years of disease duration (YDD) for patients. YDD were calculated as the difference between the age at MRI acquisition and the age of disease onset for each patient. All groups were modeled separately. To train the three CTh models, we used the following strategy: the HC model was trained with HC participants, the AD model was trained with AD patients, and the FTD model was trained with FTD patients. We introduced regional measures of both hemispheres leading to a total of 68 CTh values per subject together with time (chronological age or YDD) to train the SVR model. The overall performance of each model was assessed using the root-mean-square error (RMSE) for each CTh region (i.e., the error between estimated and real values), averaged across individuals. During training, the following SVR hyperparameters were introduced with a Grid Search, and we used a cross-validation of 5 folds with the train set (all the cross-sectional data). Models were implemented in Python version 3.10.6 (www.python.org) with the scikit-learn library [27].

### Correlation with CSF biomarkers and cognition

**Fig. 1:**
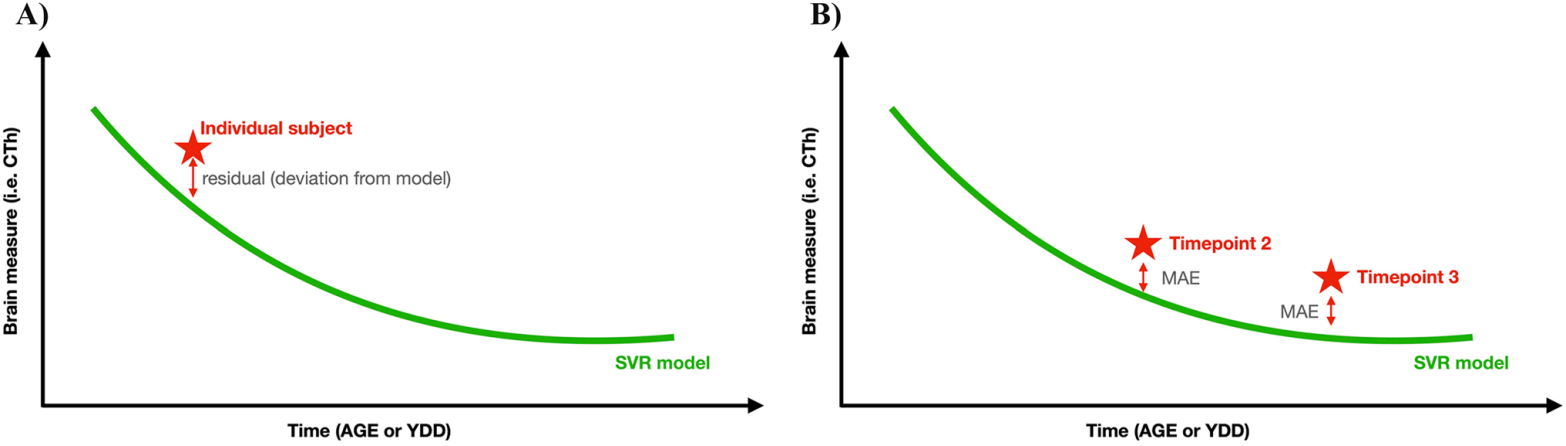
Cartoon representation of the methodology used. **A** CTh model (green line) and estimation of a sample individual’s deviation from the model. **B** Methodology used to test the model with real follow-up visits. *YDD* years of disease duration, *SVR* Support Vector Regression, *MAE* mean absolute error

We calculated the individual residuals of the model for each region and disease (Fig. 1A). We obtained deviations from the HC-derived model for each patient, and we computed the correlation between these residuals and Mini-Mental State Examination (MMSE) scores and YDD. Then, we estimated the deviations from its own disease model and computed the correlation between the obtained residuals and available CSF biomarkers levels (NfL and 14-3-3) and MMSE scores. The FTD group was first studied as a whole and further divided into subgroups (bvFTD, svPPA, and nfvPPA). A *p* value < 0.05 was set as the threshold for significance in the correlations after applying the correction for multiple comparisons for the number of regions with Benjamini–Hochberg for all the analyses. Correlations were implemented in R language, version 4.2.1 (https://www.r-project.org).

### Prediction of longitudinal changes in CTh maps 

The longitudinal data were used as unseen test samples to obtain individual predictions using the group-specific CTh models. Then, we calculated the mean absolute error (MAE) between predicted and real measures for each region (Fig. 1B). We also studied the different subgroups of FTD patients (bvFTD, nfvPPA, and svPPA). These comparisons were also implemented in R version 4.2.1.

## Results

### Sample demographics

**Fig. 2:**
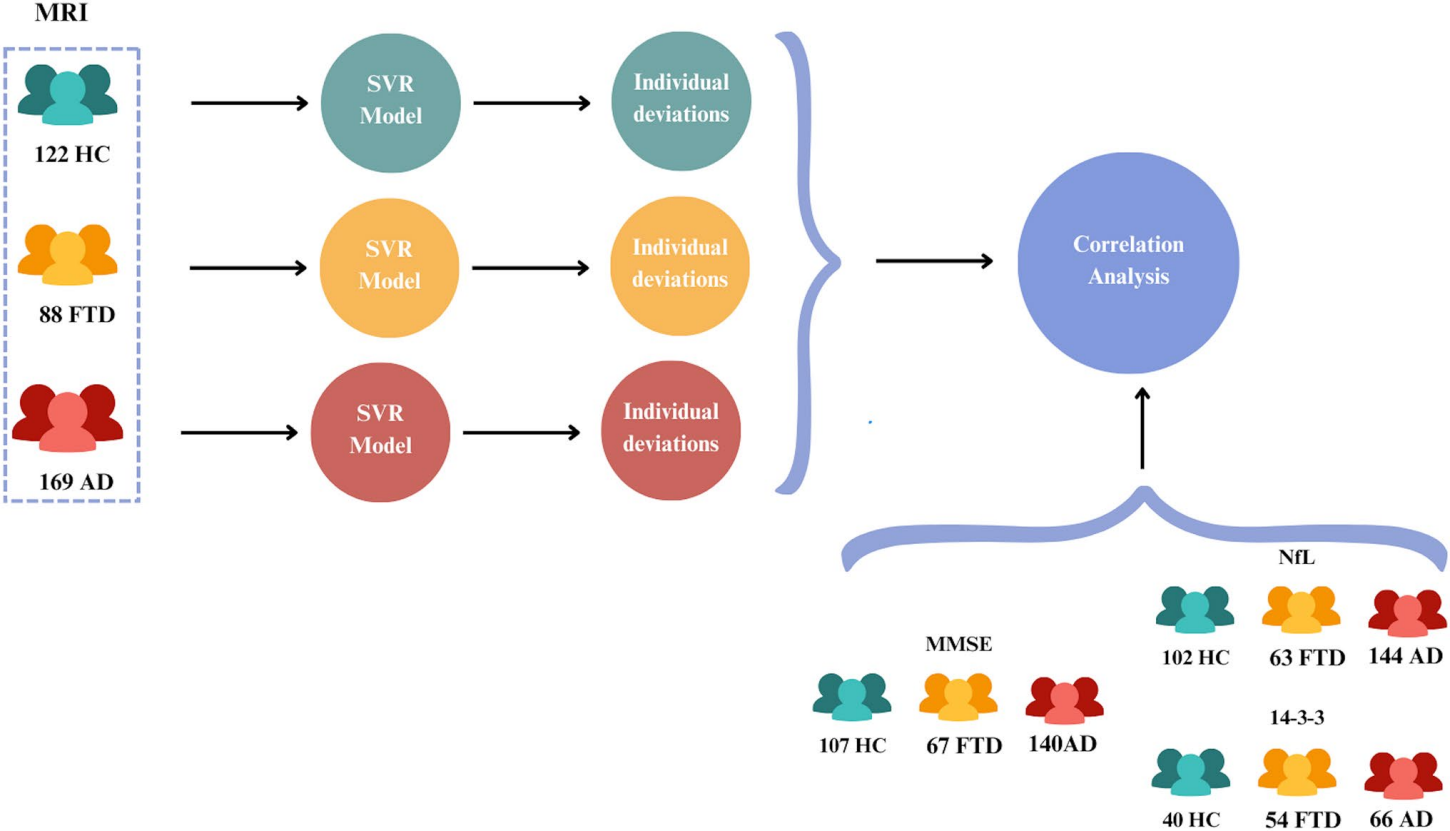
Study workflow, including the number of subjects for each group and biomarker

Of the 379 subjects included in the analyses, 169 were AD patients (*n = *29, 2 years of follow-up), 88 were FTD (*n = *27, with 2 years of follow-up, and *n = *9, with 4 years of follow-up), and 122 were HC (*n = *88, with 2 years of follow-up, and *n = *49, with 4 years of follow-up). The FTD group included 47 bvFTD patients (*n = *11, with 2 years of follow-up, and *n = *4, with 4 years of follow-up), 22 svPPA patients (*n = *9, with 2 years of follow-up, and *n = *3, with 4 years of follow-up), 17 nfvPPA patients (*n = *6, 2 years of follow-up), and 2 unspecified PPA patients (*n = *1, 2 years of follow-up). Of the 309 subjects with available NfL levels, 144 were AD patients, 63 were FTD patients, and 102 were healthy controls. For the 14-3-3 samples, we had 66 AD patients, 54 FTD patients, and 40 healthy controls with available data for our analysis. Figure 2 shows a schema of the samples available for each analysis. Demographic information and group statistics are shown in Table 1 and in Supplementary Material. In summary, as expected, CSF biomarkers levels and MMSE scores showed significant differences between groups (corrected *p* value < 0.05). Notably, the time between scans was modeled inside the models to control the potential differences between individuals.

**Table 1:** Group summaries are given as each measure's mean and standard deviation

	HC	FTD	AD	HC-FTD *p* value	HC-AD *p* value	FTD-AD *p* values
N at first MRI	122	88	169	–	–	–
N at second MRI	88	27	29	–	–	–
N at third MRI	49	9	–	–	–	–
Sex at first MRI, men/women	47/75	43/45	65/104	0.24	1.0	0.24
Sex at second MRI, men/women	29/59	14/13	12/17	0.33	0.59	0.59
Sex at third MRI, men/women	15/34	5/4	–	0.25	–	–
Age at first MRI, years (SD)	61.9 (9.8)	63.6 (8.6)	63.3 (9.4)	0.29	0.29	0.79
Years of disease at first MRI, years (SD)	–	3.3 (2.1)	2.9 (1.8)	–	–	0.10
N MMSE	107	67	140	–	–	–
Mean MMSE (SD)	28.7 (1.2)	24.5 (4.5)	22.3 (4.5)	**5.4e-12**	** < 2.0e-16**	**7.9e-5**
N NfL	102	63	144	–	–	–
Mean Nfl, pg/mL (SD)	564.3 (312.7)	2358.9 (1765.9)	1122.8 (616.9)	** < 2.0e-16**	**3.9e-6**	** < 2.0e-16**
N 14–3-3	40	54	66	–	–	–
Mean 14–3-3, pg/mL (SD)	2637.3 (734.7)	4381.4 (1931.6)	6458.4 (591.3)	**0.037**	**1.2e-5**	**0.0076**

*HC* healthy controls, *AD* Alzheimer’s disease, *FTD* frontotemporal dementia, *MMSE* Mini-Mental State Examination, *NfL* neurofilament light chain

Differences between groups are calculated using Fisher Test for sex or the ANOVA Test for the rest of the variables. Significant group differences are highlighted in bold, pairwise differences were measured with a Benjamini–Hochberg correction *p* value)

### CTh models with time and correlations with disease duration and cognition

**Fig. 3:**
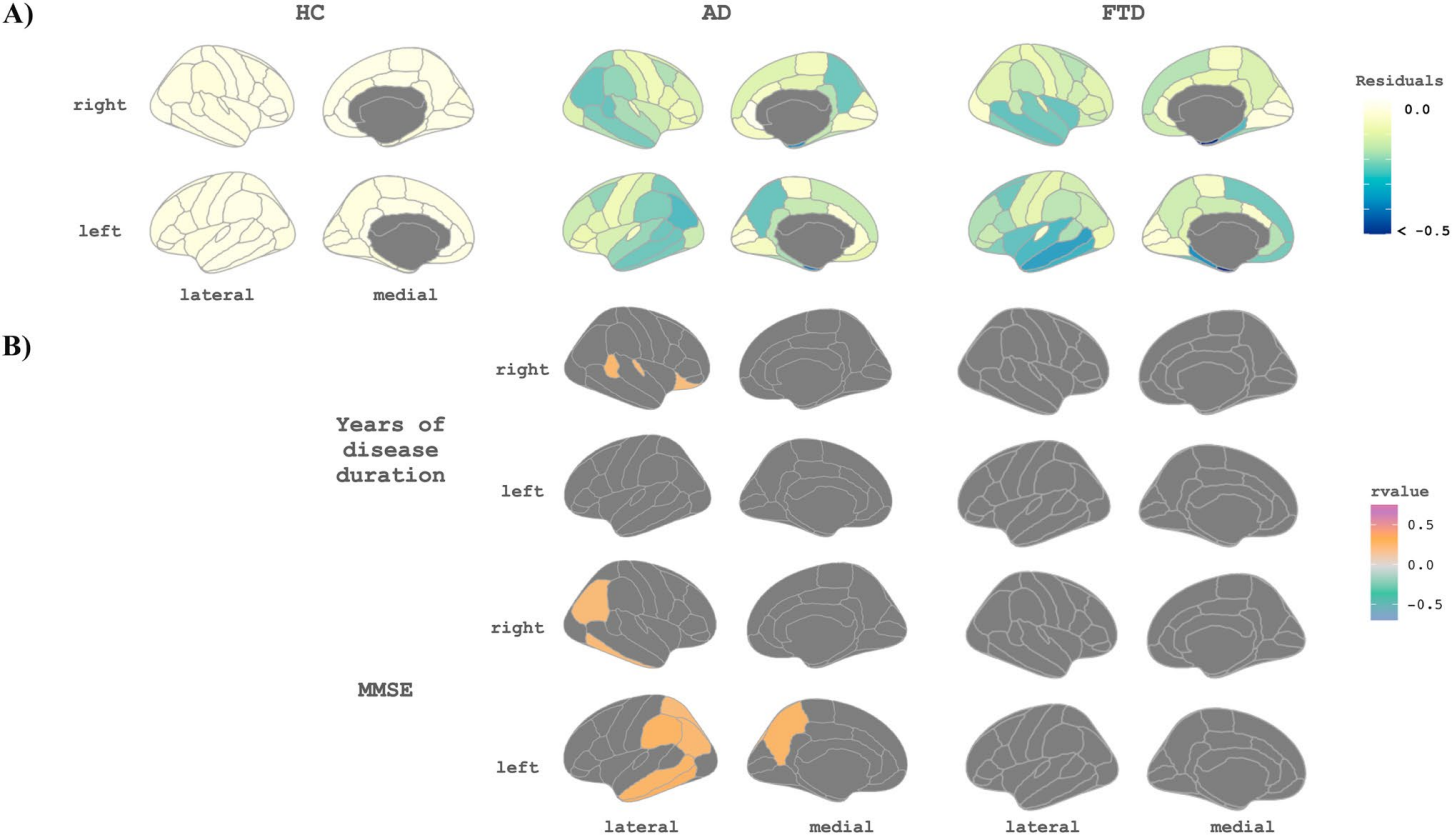
**A** Maps of the residual values for each group against the proposed healthy subjects’ model for cortical thickness. **B** Maps of correlations between residuals against the HC model of cortical thickness and clinical biomarkers in HC, AD, and FTD subjects. Only significant regions are shown, the threshold was set at 0.05 with a *p* value adjusted with multiple comparisons of all the CTh regions. *HC* healthy controls, *AD* Alzheimer’s disease, *FTD* frontotemporal dementia, *MMSE* Mini-Mental State Examination

We obtained a CTh model for HC with chronological age. The mean RMSE of the model was 0.024. Then, we obtained individual deviations for HC, AD, and FTD subjects from this HC model using the residuals (Fig. 3A). HC subjects showed values around zero, meaning that the observed and the estimated values are closer for most subjects, indicating a good fit. We obtained high residual values (absolute values between 0.01 and 0.77) for the AD and FTD, indicating high deviations from the HC-defined model (Fig. 3A). The negative residuals indicate that the real CTh values of AD and FTD subjects were lower than those estimated by the HC model. In AD, higher deviations from the HC model were only associated with higher YDD in the regions: right bankssts, right transverse temporal, right lateral orbitofrontal, and right frontal pole and with MMSE in temporal and parietal regions (corrected *p* value < 0.05, Fig. 3B). No significant correlations were identified in FTD.

### CTh models with years of disease duration and correlations with CSF biomarkers and cognition

**Fig. 4:**
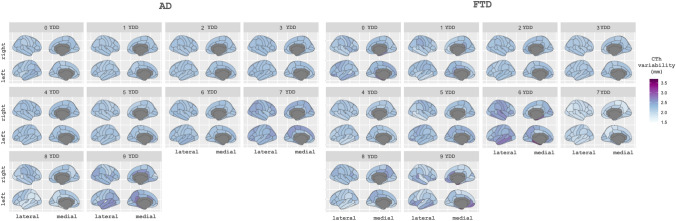
Pattern of variability of the cortical thickness estimation for each brain region according to years of disease (0–9 years) for each disease group. *AD* Alzheimer’s disease, *FTD* frontotemporal dementia, *CTh* cortical thickness, *YDD* years of disease duration

**Fig. 5:**
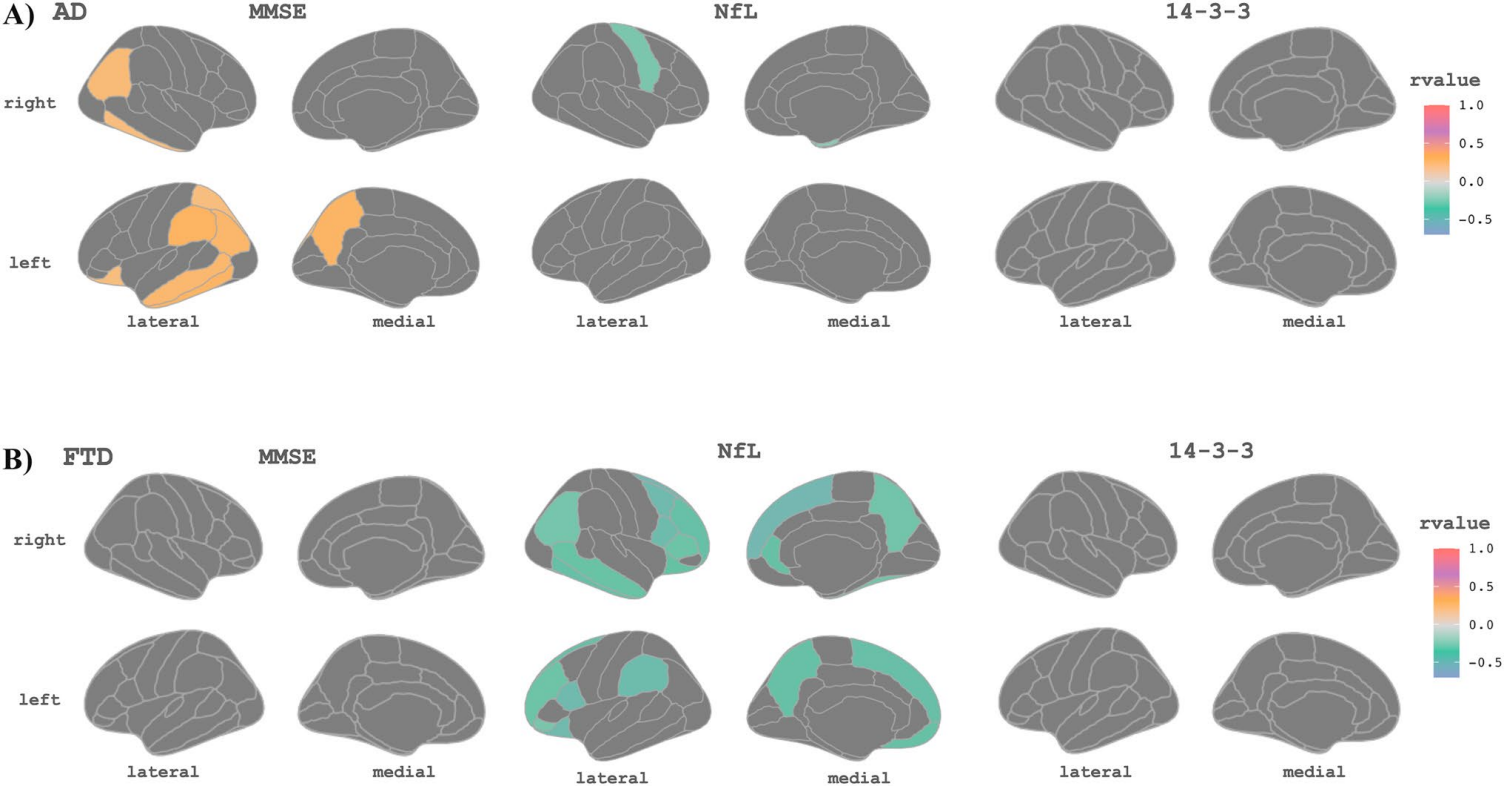
Maps of correlations between individual residuals for the group-specific cortical thickness model and cerebrospinal fluid biomarkers and MMSE scores for AD and FTD participants. Only significant regions are shown. The threshold was set at 0.05 with a *p* value adjusted with multiple comparisons of all the CTh regions. **A** Correlations within the AD group. **B** Correlations within the FTD group. *AD* Alzheimer’s disease, *FTD* frontotemporal dementia, *MMSE* Mini-Mental State Examination, *NfL* neurofilament light chain

We estimated CTh models for AD and FTD using YDD in the temporal axis. We obtained estimations of CTh in a span from 0 to 9 YDD, and we calculated the individual variations between each individual point and the estimation. The maps of variability across regions and time are shown in Fig. 4. The mean individual deviation from the model (RMSE values) for these models was: 0.08 for FTD and 0.04 for AD. We also calculated the mean values by YDD subgroups (see Supplementary Material). We studied the correlation between individual residuals from their disease model and individual CSF-NfL and CSF-14-3-3 levels and MMSE scores. In AD, the residuals of the right precentral and right entorhinal regions had a significant negative correlation with NfL levels (corrected *p* value < 0.05, Fig. 5A). The residuals of the temporal and parietal lobes positively correlated with MMSE scores, so participants with higher residual values correspond to participants with higher MMSE scores (corrected *p* value < 0.05, Fig. 5A). We did not find any correlation between 14-3-3 levels and the residuals for AD patients. In FTD, we found significant negative correlations (*p* value < 0.05, corrected) between NfL values and the residuals of the model in regions of the frontal, temporal, and parietal lobes (Fig. 5B). For 14-3-3 levels and MMSE scores, we did not find significant results. When we studied the different FTD variants, we found a negative correlation between NfL and individual residuals for bvFTD in frontal, temporal, and parietal regions (corrected *p* value < 0.05, see Supplementary Material). For the other variants (svPPA and nfvPPA), we did not find any correlation with NfL. However, high caudal middle frontal right region deviations were associated with high MMSE scores for the nfvPPA patients (corrected *p* value < 0.05, see Supplementary Material). As before, 14-3-3 levels showed no significant correlation for bvFTD, svPPA, or nfvPPA. These correlations remained significant when age was included as a covariate.

### CTh prediction of follow-up visits

**Fig. 6:**
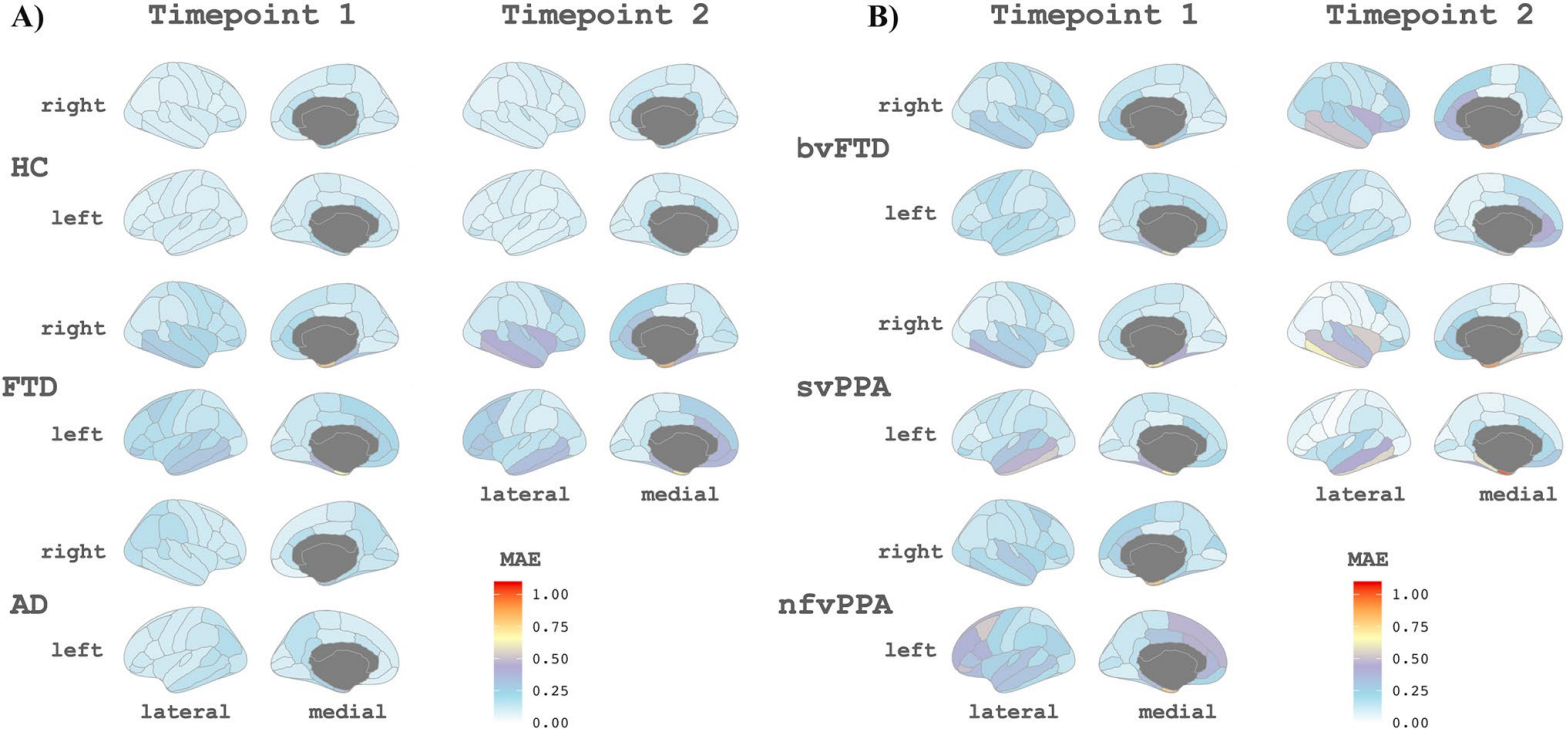
**A** Maps of the mean absolute error values of the predictions for the longitudinal data for each region for the HC, AD, and FTD groups. **B** Maps of the mean absolute error values of the predictions for the longitudinal data for the different FTD variants predicted with the FTD model. *HC* healthy controls, *AD* Alzheimer’s disease, *FTD* frontotemporal dementia, *bvFTD* behavior frontotemporal dementia, *svPPA* semantic variant primary progressive aphasia, *nfvPPA* nonfluent variant primary progressive aphasia, *MAE* mean absolute error

Finally, we explored whether the predictive CTh models trained with the baseline data could be used to predict future real CTh values. For that, we calculated the mean absolute error (MAE) values contrasting the real values (Fig. 6) at each longitudinal time point with their predicted disease model values. Also, we estimated the MAE (computed internally within the model data) for the baseline visit to use as a reference. For HC and AD (Fig. 6A), the predictive model trained with baseline data allows the prediction of future visits with low error, as the mean MAE were 0.12 and 0.15, respectively, for all the time points. For FTD (Fig. 6A), the 2 years of follow-up visit could be predicted reasonably well (mean MAE 0.20 at baseline and 0.22 at 2 years), however, the model could not present an optimal prediction for the 4 years of follow-up visit (mean MAE 0.25). We repeated the analysis with FTD data divided into subgroups (Fig. 6B), (bvFTD, nfvPPA, and svPPA). Despite the limited sample size, we found results like the whole FTD group for bvFTD and svPPA. For the nfvPPA patients (only data at 2 years of follow-up), we find the worst results in terms of predictive accuracy. In the Supplementary Material, we specify the mean MAE for each group and subgroup.

## Discussion

In this work, we implemented group-specific models of whole-brain CTh according to chronological age for HC and YDD for AD and FTD patients using SVR. We modeled CTh changes with time, and we demonstrated these models’ capability to identify individual variations as deviations from the norm. We studied individual deviations from the model defined for HC and from the disease-specific models. In AD, individual deviations from the HC-defined model correlated with YDD and MMSE scores. Using the disease-specific models, we found a significant inverse correlation between CSF-NfL levels and the deviation from the model in most of the brain regions for FTD patients, providing additional evidence of the relationship between imaging and fluid biomarkers in FTD. Furthermore, we found a positive correlation for AD between the MMSE scores and the deviation from the model in multiple regions. Finally, using real longitudinal data, we performed an exploratory study to evaluate the ability of the models described above to obtain individual predictions of future CTh values.

In healthy subjects, our model estimated whole-brain CTh using the chronological age of the subjects with residual values near zero, indicating a good fit. Several studies and multi-centric initiatives have recently focused on studying normative models with age in healthy samples [15]. Even if our healthy sample is limited compared to these studies, our results align with what has been described. In addition, we offer the applicability of such models with longitudinal data with satisfactory results. Furthermore, as deviations from normality have been widely used to assess disease severity and cognitive variability in youth [28] and in psychiatric disorders [29], we study if AD and FTD patients could be described with the previous HC model. As expected, individual AD and FTD CTh data diverged from the model defined with HC subjects. In AD, this divergence was significantly correlated with YDD and MMSE but not in FTD, suggesting that both time and general cognition impact more consistently in CTh changes compared to HC in AD. In AD, the regions of the temporal, parietal, and occipital lobes were the ones with the highest deviations from the HC model. These regions have been reported to be the most affected in patients with AD [30–33]. Therefore, our study can identify meaningful atrophy patterns using estimation models. On the other hand, the temporal and frontal lobes were the ones with the highest deviations for the FTD patients compared to the HC trend. In addition, temporal regions presented higher deviation than the AD group, depicting higher structural alterations in FTD [11, 32, 34, 35].

Given that models derived from the HC group that used chronological age showed high variability for these groups, we expected that modeling disease duration could better capture these variations. In this sense, we described CTh models in AD and FTD using YDD instead of the chronological age even though both AD and FTD individual values evidence the existence of variability within these diseases. These results complement other research highlighting these models to assess heterogeneity in neurodegenerative disorders [1, 36, 37].

We found that the individual AD deviations from the AD CTh model in the right precentral and right entorhinal inversely correlated with the NfL levels. At the regional level, the correlations between MMSE scores and the deviations from the model in AD were in the temporal and parietal lobes. In AD, MMSE has been associated with CTh [4, 33, 38]. In our case, the AD patients who deviated more from the model trend had high MMSE scores. Previous studies such as Valtteri et al. [38] showed that less CTh in frontal and temporal lobes is associated with lower MMSE scores in the AD group, which coincides with our regions and the direction of the correlation. Even if we study variability and not direct CTh measures, the regions that appear significant in the correlation analysis have high coincidence with the AD cortical pattern, which includes the temporal and parietal lobes appear [30, 31, 33, 34].

The deviations of the FTD patients from their own model presented correlations with CSF-NfL levels in the frontal, temporal, and parietal lobes. The frontal and the temporal areas are known to be the most affected in patients with FTD compared to HC [32, 34, 39]. In FTD, NfL levels have been suggested as a maker of the disease severity [40, 41]. We found that higher levels of NfL corresponded to lower residual values. Thus, patients with CTh patterns that adjusted well to the model had higher CSF-NfL levels, suggesting that the model captures the trend of neurodegeneration. Instead, lower NfL levels could correspond to patients that move further away from the FTD model, as has been described for slowly progressive FTD or non-progressors FTD [42]. Indeed, when studying the different FTD variants, we found that the main pattern was mainly driven by bvFTD, as other variants, such as PPA, showed much higher deviation. In addition, the fact that the parietal lobule emerged, which has not been reported previously, could be due to the high presence of bvFTD patients in the FTD sample. At the same time, the individual deviations in PPA patients did not present any significant correlation with NfL, suggesting that the heterogeneity in these patients' CTh pattern is not related to CSF-NfL levels. These results might provide additional support to the use of NfL levels as a marker of neurodegeneration and disease severity in FTD, paving the way for its future use as an outcome measure for clinical practice. In addition, the inexistent correlation with MMSE scores in FTD patients could be due to the fact that MMSE is not a sensitive cognitive measure in these patients, contrary to AD patients.

At the longitudinal level, we explored the application of these predictive models, trained at a cross-sectional level, to predict CTh patterns for future visits. We used the longitudinal data to know if the pseudo-longitudinal models developed with baseline data could predict the CTh values of future visits of these patients. We compared the predicted values of the longitudinal data with the real values of those visits. For healthy subjects and AD, we found that the model could predict well the CTh values at future visits. For FTD patients, the variability observed at baseline was reproduced for the longitudinal data and increased at each visit. Then, we focus on the FTD variants; the bvFTD patients fit best in future prediction model, and the nfvPPA patients were the worst. Our results align with previous studies showing that FTD is phenotypically heterogeneous [21, 22] and support that an independent predictive model should be created for each FTD phenotypic variant. Similarly, in a recent study of genetic FTD, Poos et al. [43], identified differences between the three major FTD gene mutation carriers' trajectories, suggesting that each genetic FTD subtype should also be modeled separately. The fit showed more FTD heterogeneity than AD at baseline and longitudinal levels. Overall, the proposed models could be the starting point to be able to differentiate different subtypes or dementias through the estimation of their CTh values or variabilities. Thus, further studies with a bigger cohort should study this point in detail.

Our study has some limitations. One of the major limitations of the study is the sample size. The sample size limited the generation of different CTh models for each FTD variant, especially all models at the third visit. However, the unicentric nature of the study, even if limited in the sample size, also provided homogeneity to the data acquisition. We only tested the effect of two selected neurodegeneration biomarkers in the individual residuals from their disease model. The selection of these two biomarkers was, at a certain point, arbitrary. Future studies could evaluate whether other markers could better explain the individual variability.

In conclusion, SVR provides the opportunity to generate CTh disease models to predict longitudinal changes and to study individual variability in AD, FTD, and healthy individuals and their relationships with cognitive features and biomarkers.

### Supplementary Information

Below is the link to the electronic supplementary material.

Supplementary file1 (DOCX 324 KB)

## Data Availability

The data supporting this study’s findings are available from the corresponding author upon reasonable request.
