# Supplementary material for: Cortical thickness modeling and variability in Alzheimer’s disease and frontotemporal dementia
Source: J Neurol. 2023 Nov 27;271(3):1428–38. doi: 10.1007/s00415-023-12087-1 (PMC10896866; doi:10.1007/s00415-023-12087-1)
Supplement: Supplementary file 1 — Supplementary file1 (DOCX 324 KB) [file 415_2023_12087_MOESM1_ESM.docx]

# Supplementary Material

## Sample demographics

We present the sample demographics for the different variants of frontotemporal dementia (FTD) participants in Table 1.

Table 1: Group summaries are given as each measure's mean and standard deviation. Differences between groups are calculated using Fisher Test for sex or the ANOVA Test for the rest of the variables. Significant group differences are highlighted in bold, pairwise differences were measured with a Benjamini-Hochberg correction p-value). bvFTD: behavior frontotemporal dementia, svPPA: semantic variant primary progressive aphasia, nfvPPA: nonfluent variant primary progressive aphasia, MMSE: Mini-Mental State Examination, NfL: neurofilament light chain.

|  | **bvFTD** | **svPPA** | **nfvPPA** | **bvFTD-svPPA**  **p-value** | **bvFTD-nfvPPA**  **p-value** | **svPPA-nfvPPA**  **p-value** |
| --- | --- | --- | --- | --- | --- | --- |
| **N at first MRI** | 47 | 22 | 17 | ------ | ------ | ------ |
| **N at second MRI** | 11 | 9 | 6 | ------ | ------ | ------ |
| **N at third MRI** | 4 | 3 | 0 | ------ | ------ | ------ |
| **Sex at first MRI, Men/Women** | 23/24 | 13/9 | 7/10 | 0.68 | 0.78 | 0.68 |
| **Sex at second MRI, Men/Women** | 6/5 | 6/3 | 2/4 | 0.67 | 0.67 | 0.67 |
| **Sex at third MRI, Men/Women** | 2/2 | ------ | 2/1 | 1.0 | ------ | ------ |
| **Age at first MRI,**  **years (SD)** | 63.6  (9.1) | 64.0  (8.0) | 64.0  (8.8) | 0.99 | 0.99 | 0.99 |
| **Years of Disease at first MRI,**  **years (SD)** | 3.4  (2.1) | 3.0  (2.4) | 3.6  (1.8) | 0.77 | 0.75 | 0.75 |
| **N MMSE** | 37 | 17 | 11 | ------ | ------ | ------ |
| **Mean MMSE (SD)** | 24.3  (5.0) | 24.4  (3.3) | 25.9  (4.4) | 0.93 | 0.60 | 0.60 |
| **N NfL** | 32 | 18 | 12 | ------ | ------ | ------ |
| **Mean Nfl, pg/mL**  **(SD)** | 2215.2  (1988.6) | 2167.9  (1146.1) | 2976.9  (1957.1) | 0.93 | 0.34 | 0.34 |
| **N 14-3-3** | 29 | 15 | 10 | ------ | ------ | ------ |
| **Mean 14-3-3, pg/mL**  **(SD)** | 4534.2  (2143.3) | 4585.7  (1710.2) | 3632.1  (1536.7) | 0.93 | 0.35 | 0.35 |

## CTh models with years of disease duration and correlations with CSF-biomarkers and cognition

We present the results of the mean residuals of the model grouped by baseline YDD in Table 2.

*Table 2: Mean of the CTh residuals across all regions for each model and separated by years of disease duration. AD: Alzheimer’s disease, FTD: frontotemporal dementia, and YDD: Years of disease duration.*

|  | **FTD** | **AD** |
| --- | --- | --- |
| **0 YDD, mean residual (SD)** | 0.04 (0.08) | 0.02 (0.10) |
| **1 YDD, mean residual (SD)** | 0.01 (0.14) | 0.02 (0.13) |
| **2 YDD, mean residual (SD)** | 0.01 (0.13) | 0.01 (0.12) |
| **3 YDD, mean residual (SD)** | 0.04 (0.14) | 0.01 (0.08) |
| **4 YDD, mean residual (SD)** | 0.05 (0.16) | 0.00 (0.10) |
| **5 YDD, mean residual (SD)** | 0.02 (0.21) | 0.03 (0.11) |
| **6 YDD, mean residual (SD)** | 0.13 (0.07) | 0.00 (0.11) |
| **7 YDD, mean residual (SD)** | 0.12 (0.26) | 0.06 (0.07) |
| **8 YDD, mean residual (SD)** | 0.04 (0.10) | 0.08 (0.02) |
| **9 YDD, mean residual (SD)** | 0.03 (0.25) | 0.06 (0.02) |

## Correlation with CSF-biomarkers and cognition

We studied the correlation between individual residuals from the FTD disease model and individual CSF-NfL and CSF-14-3-3 levels and MMSE scores for the FTD variants. (Figure 2).


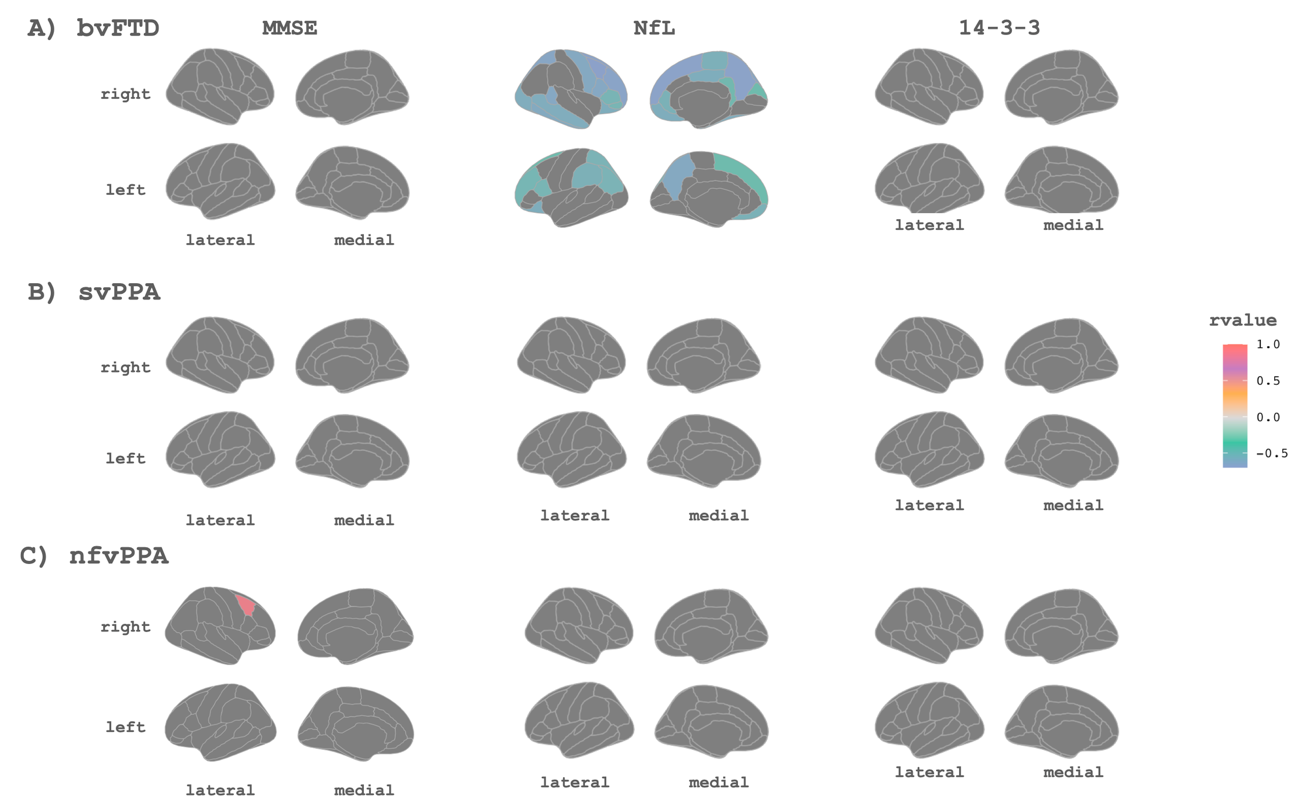


Figure 1: Maps of correlations between residuals for the cortical thickness model and cerebrospinal fluid-biomarkers and MMSE scores for the FTD variants. Only significant regions are shown. The threshold was set at 0.05 with a p-value adjusted with multiple comparisons of all the CTh regions. bvFTD: behavior frontotemporal dementia, svPPA: semantic variant primary progressive aphasia, nfvPPA: nonfluent variant primary progressive aphasia, MMSE: Mini-Mental State Examination, NfL: neurofilament light chain.

## Prediction of Cortical Thickness for the follow-up visits

We estimated the mean absolute error (MAE) for all the visits (Table 3).

*Table 3: The mean absolute error for each group. We estimated the mean for all the regions. HC: healthy controls, AD: Alzheimer’s disease, FTD: frontotemporal dementia, bvFTD: behavior frontotemporal dementia, svPPA: semantic variant primary progressive aphasia, nfvPPA: nonfluent variant primary progressive aphasia.*

|  | Baseline | Timepoint 2 | Timepoint 3 |
| --- | --- | --- | --- |
| HC | 0.12 | 0.12 | 0.12 |
| AD | 0.15 | 0.15 | ------ |
| FTD | 0.20 | 0.22 | 0.25 |
| bvFTD | 0.20 | 0.20 | 0.23 |
| svPPA | 0.20 | 0.22 | 0.23 |
| nfvPPA | 0.20 | 0.26 | ------ |
